# Reliability analysis of a sensitive and independent stabilometry parameter set

**DOI:** 10.1371/journal.pone.0195995

**Published:** 2018-04-17

**Authors:** Gergely Nagymáté, Zsanett Orlovits, Rita M. Kiss

**Affiliations:** 1 Department of Mechatronics, Optics and Mechanical Engineering Informatics, Budapest University of Technology and Economics, Budapest, Hungary; 2 Institute of Mathematics, Budapest University of Technology and Economics, Budapest, Hungary; University of Innsbruck, AUSTRIA

## Abstract

Recent studies have suggested reduced independent and sensitive parameter sets for stabilometry measurements based on correlation and variance analyses. However, the reliability of these recommended parameter sets has not been studied in the literature or not in every stance type used in stabilometry assessments, for example, single leg stances. The goal of this study is to evaluate the test-retest reliability of different time-based and frequency-based parameters that are calculated from the center of pressure (CoP) during bipedal and single leg stance for 30- and 60-second measurement intervals. Thirty healthy subjects performed repeated standing trials in a bipedal stance with eyes open and eyes closed conditions and in a single leg stance with eyes open for 60 seconds. A force distribution measuring plate was used to record the CoP. The reliability of the CoP parameters was characterized by using the intraclass correlation coefficient (ICC), standard error of measurement (SEM), minimal detectable change (MDC), coefficient of variation (CV) and CV compliance rate (CVCR). Based on the ICC, SEM and MDC results, many parameters yielded fair to good reliability values, while the CoP path length yielded the highest reliability (smallest ICC > 0.67 (0.54–0.79), largest SEM% = 19.2%). Usually, frequency type parameters and extreme value parameters yielded poor reliability values. There were differences in the reliability of the maximum CoP velocity (better with 30 seconds) and mean power frequency (better with 60 seconds) parameters between the different sampling intervals.

## Introduction

Postural stability is the necessary condition for retaining and recovering balance [1], which allows the body’s center of mass (CoM) to be kept close to the same location with respect to the body stance. Vertical posture and balancing during everyday activities requires constant retention of controls that require adequate and precise somatosensory, visual, and vestibular senses and a sufficiently accurate response of the nervous and musculoskeletal systems. Interpretation of postural stability and measurements and analyses with different methods are fundamental tools in the exploration of balancing capability disruptions and in the assessment of treatment effectiveness, since they are simple to perform, not burdensome for the subject, and require short testing times [1]. Stabilometry is the objective study of body sway during quiet standing, i.e., stance in the absence of any voluntary movements or external perturbations. Stabilometry is usually based on the analysis of the time variant center of pressure (CoP) coordinates during a bipedal or single leg stance with the eyes open or closed. CoP signals are usually measured on force plates or force distribution plates that are sampled at a certain frequency [2]. CoP can be utilized in clinical diagnostics [3] and is extensively applied in biomechanical research studies.

The movement of the CoP is influenced by many factors, such as age and gender [4], diseases [5], or measurement setups [6]. The International Society for Posture and Gait Research standardized many aspects of static stabilometry measurements in 2009 [6], but a wide variety of parameters that characterize CoP displacement is still used by researchers [5,7]. From the two-dimensional CoP coordinates acquired during the measurement interval, many CoP parameters can be derived that can be classified into time-domain and frequency-domain measures. The load distribution difference (LDD) between the two lower limbs is another sensitive stabilometry parameter that was successively used for assessing differences in the postural stability of unilaterally and bilaterally involved knee osteoarthritis patients in previous research studies [8]. This parameter could also be used in the discrimination of early medial knee osteoarthritis [9].

Many researchers have analyzed the test-retest reliability of certain CoP parameters on homogeneous groups of test subjects, e.g., on subjects with incomplete spinal cord injury [10], hip osteoarthritis [11], knee osteoarthritis [12], healthy elderly people [13], musculoskeletal disorders [14], stroke survivors [15], healthy young adults [4,16–19] or healthy subjects of various ages [20]. Most studies that focused on CoP parameter reliability following the conventional test-retest analyses required that the participants perform two sessions on different days or separated by at least one-hour time intervals [11,12,14,15,17]. Within a session, there could be multiple averaged measurements. Other studies performed multiple measurements sequentially to analyze the intrasession reliability [13,19]. Some authors established the existence of postural balance changes on different days [18] or even during the day [21,22]. The most widely used reliability measurements by these studies include the intraclass correlation coefficient (ICC), standard error of measurement (SEM), minimal detectable change (MDC) and coefficient of variation (CV), which is also known as the relative standard deviation [20]. The results of most of these studies are well summarized in a literature review by Ruhe et al. [7], while later studies continued to research the reliability of new sophisticated stabilometry parameters [19].

Many of the above reliability studies analyzed the reliability of CoP parameters with certain measurement setups, protocols and sampling conditions, while some research studied the effects of different settings on the parameter reliability. Recommendations of stabilometry standardization are based on these studies, which changed during the years in the light of newer results. While earlier, a minimum of 60-second sampling interval was recommended [23], a more recent standardization initiative recommends a minimum of 25-second sampling interval for time-distance type parameters in stabilometry [6]. However, due to the large number of CoP parameters, not every recommendation covers all parameters. Many of these parameters contain redundant information [24], such as the path length and average path velocity in the case of the standardized measurement interval [10].

To reduce the large number of CoP parameters, Nagymáté and Kiss studied many of them with correlation analysis and variance analysis in different standing conditions to select the independent CoP parameters that are sufficiently sensitive to show the differences between the different standing conditions [25,26]. The authors recommended the time-distance and frequency parameters as independent CoP parameter sets presented in Table 1. Based on the literature review, only Santos et al. [16] studied the reliability of the time and frequency domain parameters together, but only during bipedal stances. Reliability findings in a bipedal stance with the eyes opened and closed are valid not only for young, healthy persons but also for the elderly or orthopedically or neurologically altered patients [7]. Reliability analysis of CoP parameters during a single leg stance is also heavily important because the single leg stance is an important test case in the analysis of the effect of the foot structure on the postural stability [27–29]; however, CoP parameter reliability on single leg stances has been rarely studied and only on averaged values [30] in shorter (30-second) trials compared with the recommended sampling interval of 60 seconds for some studied frequency type parameters [23].

**Table 1:** Studied parameters.

Parameter name	Dimension	Description
*Time-distance parameters*		
Confidence ellipse area (CE area)	mm^2^	The area of the 95% confidence ellipse around the CoP trajectory [36].
Confidence ellipse axis ratio (CE axis ratio)	1	The ratio between the major and minor axes of the 95% confidence ellipse that describes the shape of the CoP’s trajectory expansion.
Path length	mm	The length of the total CoP trajectory during the measurement.
Maximum path velocity	mm/s	The filtered maximum distance between consecutive CoP points divided by the sampling interval.
AP-ML range ratio	1	The ratio of the largest CoP path expansions in the anteroposterior (AP) and mediolateral (ML) directions that describes the relation of the largest random errors of postural control between the two anatomical directions.
Anterior (AP+) and Posterior (AP-) maximum deviations	mm	The maximum excursions in the anterior and posterior direction relative to the average CoP point in the AP-ML plane (Fig 1)
Largest amplitude during balancing (LA)	mm	The largest continuous motion in both the AP (Fig 2) and the ML directions, which are not necessarily equal to the corresponding CoP range. This parameter is similar to the sub-movement size that was defined by [37] for targeted CoP movements.
*Frequency parameters*		
Frequency power ratios between low-medium and medium-high frequency bands (LMR, MHR)	1	Provide information about the power distribution of the postural sway in the frequency domain. The defined limits of the compared frequency bands are low- (0–0.3 Hz) medium- (0.3–1 Hz) and high frequency (1–5 Hz) bands [38].
Mean power frequency (MPF)	Hz	A weighted average frequency where the *f*_*j*_ frequency components are weighted by their *P*_*j*_ power. *M* is the number of frequency bins. MPF is calculated as proposed by Oskoei and Hu [39], according to the following equation: $MPF=\sum_{j=1}^{M}f_{j}P_{j}/\sum_{j=1}^{M}P_{j}$
Spectral power ratio (SPR)	1	The ratio of the total spectral power in the AP direction and the total spectral power in the ML direction. SPR characterizes the rate of the power distribution of the postural sway frequencies in the AP/ML directions.
*Other*		
Load distribution difference (LDD)	%	Shows the difference in the weight load on the lower limbs. This parameter is not derived from CoP motion, but it is used by the original Zebris WinPDMS software together with the CoP parameters and is proven to be very useful in biomechanical analyses [8,9].

**Fig 1:**
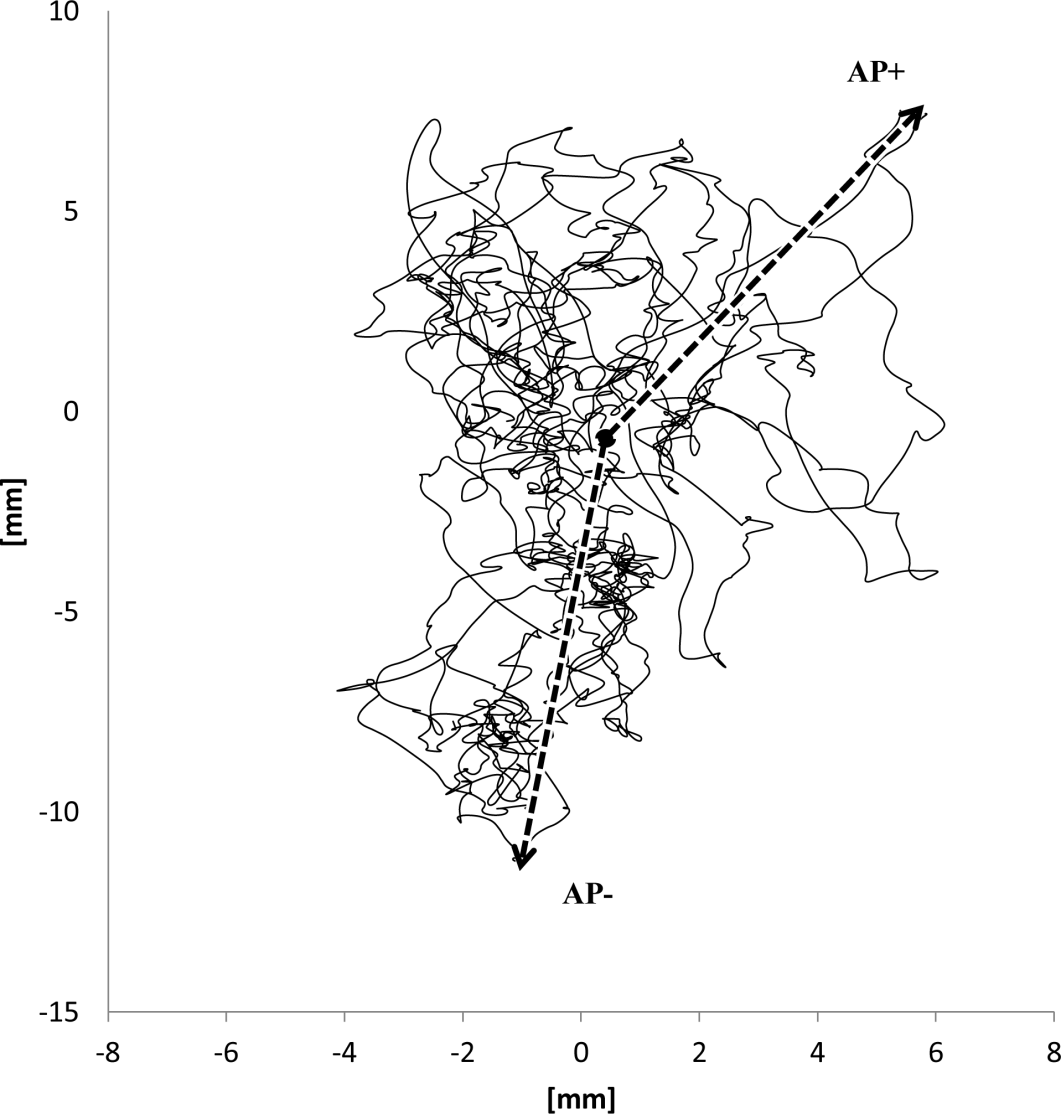
CoP anterior (AP+) and posterior (AP-) maximum deviation.

**Fig 2:**
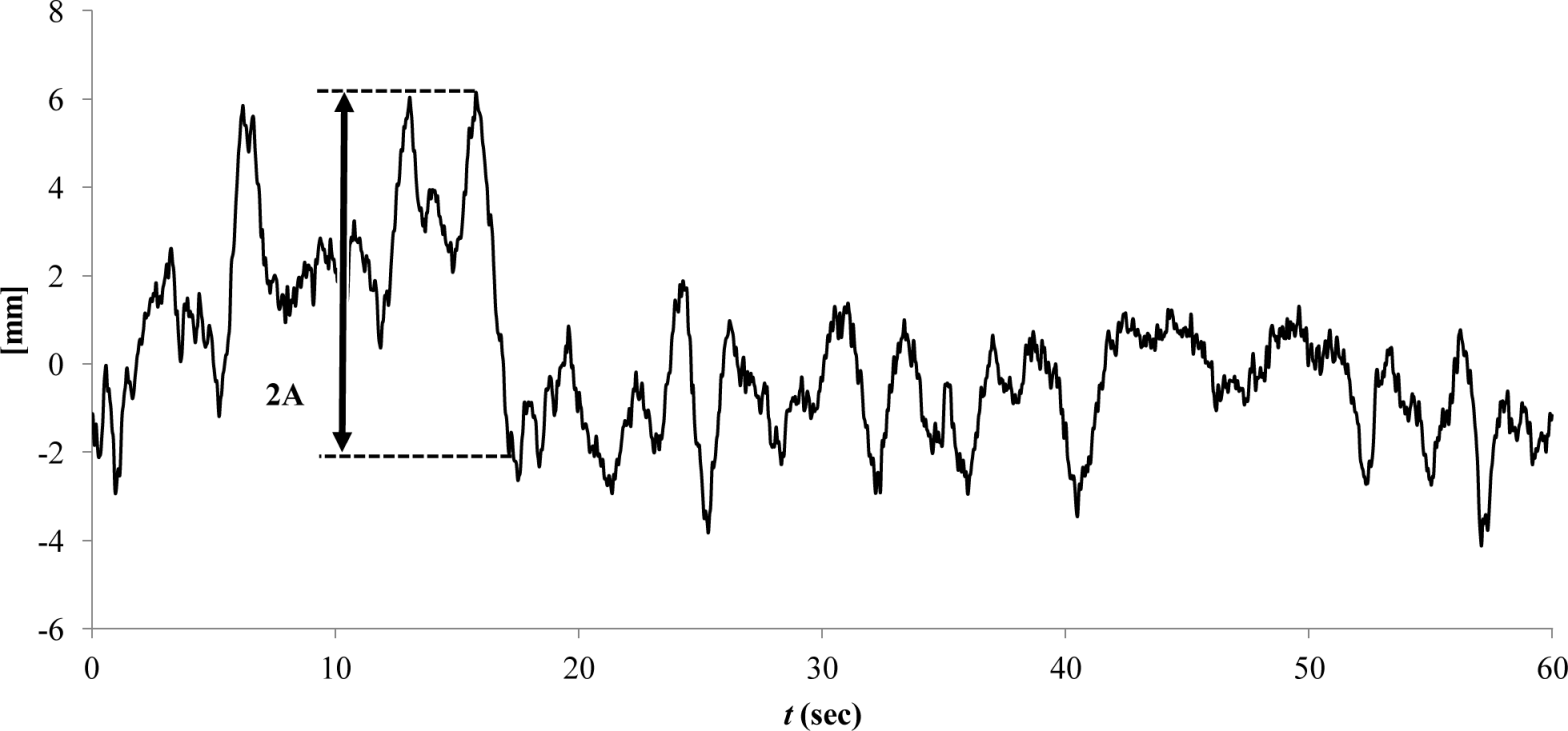
Largest amplitude (A) in the AP direction.

On the basis of the literature, the present paper aims to evaluate the test-retest reliability of the independent time-distance and frequency-based CoP parameters that are recommended as a conclusion of correlation analyses in [25,26]. The bipedal stance with the eyes open (EO), eyes closed (EC) and stance on the dominant leg (SL) were involved in our measurement protocols, and the reliability of the parameters was determined for both 30- and 60-second sampling intervals with repeated standing trials of healthy young participants on a force distribution plate. It is expected that the summary parameters (such as path length) will be more reliable in our parameter set owing to their averaging effect compared to extremity- and frequency-based parameters, which are more influenced by randomness.

## Methods

This study follows the Guidelines for Reporting Reliability and Agreement Studies [31] and the recommendations for reliability studies [32,33] and general recommendations for stabilometry studies [6,7].

### Participants

Initially, assuming some consistency, a minimum sample of 26 participants was required for observing reliability 0.6 (planning value) compared with 0.4 (minimum values) by one rater with ten trials per participant and with *α* = 0.05 (significance level) and *β* = 0.8 (statistical power) [33].

Thirty healthy subjects were included in the study (22 males, 8 females; average age: 22.0 ± 6.47 years; average body height: 1.77 ± 0.06 m; average body mass: 73.0 ± 14.76 kg, BMI: 23.16 ± 4.25). The exclusion criteria included the following: a history of vertigo or dizziness, vestibular or neurologic disorders, uncorrected visual problems, sustained lower extremity injuries, spinal disorders, use of medications that influence the balance system, hearing loss, and acute/chronic ear infections.

### Ethical approval

The participants received detailed oral and written information about the risks and benefits of the study. Each participant gave signed informed consent and was given the opportunity to withdraw from the study at any time. The study was approved by the Hungarian National Science and Research Ethics Committee (114/2004).

### Equipment

Stabilometry was performed on a Zebris FDM-S multifunctional force distribution measuring plate (320 mm × 470 mm measuring surface with 1504 load cells) (ZEBRIS GmbH, Isny, Germany) in the motion analysis laboratory of the Department of Mechatronics, Optics and Mechanical Engineering Informatics of the Budapest University of Technology and Economics (Hungary). During the measurements, the room was silent, and no other activities were performed in the room that could distract the measurement. The room temperature was comfortable for the participant’s attire.

### Procedure

The measurement procedure has been registered and is accessible in https://dx.doi.org/10.17504/protocols.io.ns6dehe. Before performing stabilometry, the basic anthropometrical data for all participants were registered. Specifically, the body height was measured and recorded in cm; and the body mass was measured to the nearest 0.1 kg with an electronic weight scale with the participants wearing shorts and a T-shirt. During the measurements, the participants were barefoot.

The studied stance types were bipedal stance with the eyes open (EO) or eyes closed (EC) and stance on the dominant leg (SL). In each session, the order of the stance types was randomized and was continued successively without rest. Before the test, the dominant side of each participant was determined by a balance recovery test. The perturbation was a nudge from the tester applied to the subject at the midpoint between the scapulae from directly behind the subject and sufficient to require the participant to respond by taking a step. The leg that the subject used to recover balance was considered to be the dominant leg [34]. The investigated subjects were positioned in bipedal standing, with the distance between the 2 ankle joint centers equal to the distance between the right and left anterior superior iliac spines. Both limbs were in full knee extension, the heels were aligned in a line, and the feet were parallel and faced forward, with arms resting by the sides. During the stance on the dominant leg, the dominant leg was in full knee extension, and the non-dominant leg was flexed at 90 degrees. Since a 60 second single leg standing trial is challenging, in case the subject had to put down the other leg or showed substantial arm or whole body movements, the measurement was aborted and repeated. This event was rare because the participants were young, healthy individuals capable of performing a 60-second single leg stance. The exact foot placement was maintained during the trials and was visually controlled since its changes could affect the stabilometry parameters [35]. During stances with the eyes open (EO and SL), the eyes were focused on the wall at eye level 5 meter from the subject. The measurements started after the initial transients of the subject’s self-adjustment. The adjustment time was usually approximately 10 seconds, but at a minimum 5 second wait after the subject stood on the platform. The pressure distribution under the feet was monitored by the measuring person on the computer screen to validate if the subject was standing still. Each measurement was conducted by the same person (GN).

Each session was repeated ten times. Between sessions, there were 1- to 5-min resting periods according to the needs of the subject, where talking and sitting down was permitted to avoid the effects of boredom and fatigue. Subjects were asked to walk in the room between sessions, and they had to be standing for at least half a minute before the next session started to avoid dizziness caused by a possible drop in the blood pressure owing to the sudden change of stance to the standing position.

The sampling frequency of the force distribution plate was set to 100 Hz. The sampling duration was set to 60 seconds, as suggested by Carpenter et al. [23], from which the first 30 seconds was used in the 30-second measurement intervals.

### Measured and assessment parameters

Force distribution data was recorded by the Zebris WinPDMS processing software (v1.2). Further data processing and calculation of the CoP parameters was conducted on exported raw measurement data in a custom application written in LabVIEW v2013 (National Instruments Inc., Austin, Texas). The calculated instantaneous CoP coordinates were filtered by a Butterworth low-pass digital filter with a cut-off frequency of 10 Hz, as recommended by Ruhe et al. [7]. From the CoP position signals, a power spectrum was obtained using the Fast Fourier Transformation (FFT) with a Hanning filtering window. The acquired frequency resolution was 0.016 Hz. The studied parameters are described in Table 1.

### Statistical analyses

The combinations of the 30 healthy participants and the three stance types are considered to be our test cases for reliability assessment, with ten repetitions in each case, which results in ninety test cases and 900 individual measurements. The reliability in each stance type for the 30- and 60-second measurement interval was evaluated by traditional indicators such as ICC, SEM and MDC. The 30 second interval was the first half of the 60 second measurement. Evaluation of the ICC was based on the recommendation of Fleiss [40], and evaluation of SEM is according to Laroche [11]. ICC(2,1) was chosen to assess the reliability using a two-way random effect model with absolute agreement for single measures [41].

ICC often results in wide confidence intervals for its results. The values of SEM and MDC are based on the ICC, and therefore, they cannot complement the ICC for reliability analysis sufficiently. Thus, the covariance of variation (*CV*) (the other term is the relative standard deviation) was also determined within subjects as the ratio of the standard deviation (*σ*) to the mean (*𝜇*), as in (1):

$$CV=\frac{\sigma }{\mu }.$$ (1)

As Atkinson and Nevill [42] describes, CV is not suitable for all types of parameters, such as parameters whose value distributes around zero (e.g., LDD). They also clarify that the calculated mean CV does not reflect the repeated test error for all individuals, but only the ‘average individual’. Correspondingly, the CV compliance rate (CVCR) was considered to extend the ICC-based analyses and to measure the ratio of the test cases in which the CV is below a given value. Ruhe et al notes in their review that CV ≤ 0.33 is a commonly used cutoff value in the interpretation of ‘acceptable’ CV [7]. As a rule of thumb, this cutoff value for the CVCR was set to 30%. The newly introduced CVCR reliability measure was defined as follows:

$$CVCR=\frac{n_{CV<30\%}}{n}$$ (2)

where *n*_*cv<30%*_ is the number of test cases in which CV ≤ 30%, and n is the total number of test cases.

The data analysis for this paper was generated using the Real Statistics Resource Pack software (Release 5.4), Copyright (2013–2018) Charles Zaiontz (www.real-statistics.com).

## Results

Thirty subjects performed ten consecutive standing trials using three stance types. No measurements had to be excluded. The results of the reliability analysis can be found in Table 2 for the eyes open condition, Table 3 for closed eyes and Table 4 for the single leg stance. Each table demonstrates the reliability variables for each of the CoP parameters summarized in Table 1 in the 30-second and 60-second measurement intervals. The ICC values are usually consistent in the 30- and 60-second intervals. The path length consistently yields the best ICC values in all configurations (> 0.7 during each condition except for EO 30 seconds, where 0.67). The LDD consistently yields fair-to-good reliability, with ICC 0.49–0.54 in both bipedal stances. The CE area yielded fair-to-good reliability in most cases. The LA parameters yielded mostly fair-to-good reliability in the 60-second measurements, while poor when only the first 30 seconds were analyzed. The ICC of AP LA was fair to good in each of the stance conditions with the 60-second measurement interval. The MPF parameters showed fair-to-good reliability in the EC and SL measurements, except for AP MPF in the 30-second SL condition. Usually, the other frequency type parameters showed poor reliability based on the ICC. The maximum CoP velocity parameter behaved differently, as it showed better (fair to good) reliability during the 30-second intervals in both bipedal stances, while it showed poor reliability with the 60-second periods. The maximum velocity has poor reliability in both SL conditions.

**Table 2:** Reliability measures with eyes open bipedal stance for 30-second and 60-second measurement intervals.

	EO 30 second					EO 60 second				
	ICC	SEM	95% MDC	CV [%]	CVCR [%]	ICC	SEM	95% MDC	CV [%]	CVCR [%]
CE axis ratio	0.36 (0.23–0.53)	0.94 (42.1%)	2.6	34.22	53.33	0.22 (0.12–0.38)	0.9 (42.5%)	2.49	34.78	50
CE area	0.39 (0.26–0.56)	67.16 (57.8%)	186.15	53.65	6.67	0.43 (0.3–0.6)	65.44 (59.3%)	181.39	49.33	3.33
Path length	0.67 (0.54–0.79)	61.81 (19.2%)	171.32	15.86	93.33	0.75 (0.64–0.85)	100.74 (16.3%)	279.24	13.63	93.33
Maximum velocity	0.43 (0.3–0.6)	32.17 (45.5%)	89.16	35.1	40.0	0.13 (0.05–0.26)	192.93 (174.1%)	534.77	58.18	36.67
AP-ML range ratio	0.2 (0.11–0.35)	0.67 (43.7%)	1.87	38.93	30.0	0.23 (0.13–0.39)	0.64 (40.8%)	1.77	37.52	30
LDD	0.49 (0.36–0.65)	7.41 (72.2%)	20.54	61.66	6.67	0.54 (0.4–0.69)	7.21 (72.2%)	19.98	62.79	13.33
AP LA	0.31 (0.19–0.48)	6.19 (42.5%)	17.17	34.56	43.33	0.46 (0.32–0.62)	6.41 (37.2%)	17.76	31.25	66.67
ML LA	0.33 (0.21–0.5)	5.59 (52.7%)	15.5	36.44	43.33	0.37 (0.24–0.54)	5.9 (48.8%)	16.36	35.79	46.67
AP+	0.21 (0.11–0.36)	7.14 (50.5%)	19.8	37.04	43.33	0.23 (0.13–0.39)	8.86 (49.8%)	24.55	36.33	53.33
AP-	0.35 (0.22–0.52)	5.05 (36.7%)	13.99	31.87	50.0	0.15 (0.06–0.29)	14.82 (82.7%)	41.09	38.48	66.67
AP MPF	0.27 (0.16–0.44)	0.08 (40.3%)	0.21	38.61	26.67	0.33 (0.21–0.5)	0.06 (41.3%)	0.16	39.75	20
ML MPF	0.22 (0.12–0.37)	0.11 (41.7%)	0.3	41.46	16.67	0.3 (0.18–0.47)	0.09 (44.7%)	0.25	44.39	20
SPR	0.13 (0.05–0.26)	4.23 (115.1%)	11.72	83.34	0	0.21 (0.11–0.36)	4.63 (116.2%)	12.83	79.41	0
AP LMR	0.22 (0.12–0.37)	7.55 (88.7%)	20.93	74.82	3.33	0.23 (0.12–0.38)	11.73 (91.6%)	32.5	67.97	0
AP MHR	0.2 (0.1–0.35)	6.53 (59.2%)	18.11	51.22	0	0.36 (0.23–0.53)	5.61 (51.6%)	15.55	41.99	13.33
ML LMR	0.15 (0.07–0.29)	8.5 (146.7%)	23.57	87.44	0	0.15 (0.07–0.29)	12.66 (144.3%)	35.1	80.65	0
ML MHR	0.39 (0.26–0.55)	4.97 (53.4%)	13.78	44.54	3.33	0.42 (0.29–0.58)	4.5 (48.6%)	12.46	42.15	23.33

**Table 3:** Reliability measures with the eyes closed bipedal stance for 30-second and 60-second measurement intervals.

	EC 30 second					EC 60 second				
	ICC	SEM	95% MDC	CV [%]	CVCR [%]	ICC	SEM	95% MDC	CV [%]	CVCR [%]
CE axis ratio	0.15 (0.07–0.3)	0.93 (42.9%)	2.57	32.51	53.33	0.17 (0.08–0.32)	0.85 (40.3%)	2.36	32.2	56.67
CE area	0.42 (0.29–0.59)	66.99 (49.3%)	185.68	43.83	20.0	0.39 (0.26–0.56)	75.41 (58.1%)	209.03	48.02	13.33
Path length	0.71 (0.6–0.82)	59.24 (15.8%)	164.2	14.14	100	0.74 (0.63–0.84)	109.88 (15.2%)	304.58	13.23	100
Maximum velocity	0.48 (0.35–0.64)	24.07 (33.1%)	66.71	27.44	66.67	0.08 (0.01–0.2)	279.33 (213.6%)	774.27	62.1	43.33
AP-ML range ratio	0.36 (0.23–0.52)	0.6 (34.6%)	1.66	32.44	46.67	0.42 (0.29–0.59)	0.54 (31.8%)	1.51	31.44	43.33
LDD	0.51 (0.38–0.67)	6.15 (69.9%)	17.05	64.24	3.33	0.52 (0.39–0.68)	5.86 (67%)	16.23	61.57	3.33
AP LA	0.36 (0.23–0.53)	6.69 (36.3%)	18.54	30.73	50.0	0.49 (0.35–0.65)	7.1 (32.7%)	19.68	27.01	73.33
ML LA	0.4 (0.27–0.56)	5.42 (45.4%)	15.03	34.99	46.67	0.48 (0.34–0.64)	5.78 (41.5%)	16.03	34.55	46.67
AP+	0.35 (0.23–0.52)	5 (32.4%)	13.86	29.23	63.33	0.29 (0.17–0.45)	8.18 (40.7%)	22.68	33.2	36.67
AP-	0.34 (0.22–0.51)	5.74 (36.2%)	15.91	31.12	53.33	0.11 (0.04–0.24)	16.64 (80%)	46.13	39.45	60
AP MPF	0.42 (0.29–0.58)	0.07 (32.8%)	0.21	32.58	43.33	0.43 (0.3–0.6)	0.06 (34.1%)	0.16	34.38	30
ML MPF	0.43 (0.3–0.6)	0.11 (36.6%)	0.29	36.27	36.67	0.4 (0.27–0.57)	0.09 (39.6%)	0.24	39.67	16.67
SPR	0.33 (0.21–0.5)	3.73 (80.1%)	10.33	70.48	0	0.36 (0.23–0.53)	3.34 (73.4%)	9.26	62.87	0
AP LMR	0.08 (0.02–0.2)	6.07 (108.3%)	16.82	79.06	0	0.2 (0.11–0.35)	6.25 (74.8%)	17.32	62.96	3.33
AP MHR	0.28 (0.17–0.45)	8.46 (67.7%)	23.45	51.85	0	0.36 (0.23–0.53)	5.76 (49.1%)	15.96	42.27	10
ML LMR	0.18 (0.09–0.33)	4.56 (108%)	12.64	82.75	0	0.24 (0.13–0.4)	6.8 (102.3%)	18.86	74.72	3.33
ML MHR	0.34 (0.22–0.51)	5.93 (56.1%)	16.43	49	3.33	0.46 (0.33–0.63)	4.64 (44.4%)	12.87	40.51	20

**Table 4:** Reliability measures with an eyes closed bipedal stance for 30-second and 60-second measurement intervals.

	SL 30 second					SL 60 second				
	ICC	SEM	95% MDC	CV [%]	CVCR [%]	ICC	SEM	95% MDC	CV [%]	CVCR [%]
CE axis ratio	0.19 (0.09–0.34)	0.92 (50.2%)	2.55	27.31	70.0	0.25 (0.14–0.41)	0.9 (49.4%)	2.5	22.89	90
CE area	0.58 (0.45–0.72)	93.63 (34%)	259.53	31.64	53.33	0.5 (0.36–0.66)	83.55 (30.8%)	231.58	26.65	73.33
Path length	0.73 (0.62–0.83)	167.8 (16.8%)	465.11	15.78	96.67	0.82 (0.73–0.89)	257.92 (13.4%)	714.92	12.74	100
Maximum velocity	0.22 (0.12–0.38)	91.44 (46.6%)	253.45	32.8	60.0	0.11 (0.04–0.24)	204.76 (78.2%)	567.57	44.8	43.33
AP-ML range ratio	0.24 (0.13–0.4)	0.6 (38.2%)	1.65	26.88	73.33	0.29 (0.17–0.45)	0.57 (34.1%)	1.59	25.03	76.67
LDD	not applicable					not applicable				
AP LA	0.29 (0.18–0.46)	8.76 (34.3%)	24.27	29.92	56.67	0.4 (0.27–0.57)	9.91 (31.9%)	27.46	28.28	66.67
ML LA	0.3 (0.18–0.47)	5.13 (25.3%)	14.21	24.17	80.0	0.25 (0.14–0.41)	6.72 (29%)	18.62	22.84	86.67
AP+	0.34 (0.22–0.51)	5.5 (23.6%)	15.26	21.54	86.67	0.26 (0.15–0.42)	9.91 (34.1%)	27.46	25.36	83.33
AP-	0.23 (0.13–0.39)	8.45 (35%)	23.41	28.53	63.33	0.31 (0.19–0.47)	9.65 (33.1%)	26.75	27.22	70
AP MPF	0.24 (0.13–0.39)	0.12 (35.4%)	0.33	34.56	50.0	0.44 (0.3–0.6)	0.08 (31.6%)	0.23	32.75	50
ML MPF	0.46 (0.33–0.62)	0.14 (25.1%)	0.38	25.3	70.0	0.55 (0.41–0.7)	0.1 (23%)	0.29	22.7	80
SPR	0.12 (0.05–0.26)	6.89 (231.1%)	19.09	58.82	10.0	0.16 (0.07–0.3)	5.16 (164.8%)	14.31	51.37	16.67
AP LMR	0.2 (0.1–0.35)	3.6 (93.7%)	9.99	67.96	0	0.38 (0.25–0.54)	3.3 (64.6%)	9.16	51.18	3.33
AP MHR	0.47 (0.34–0.64)	1.9 (44.8%)	5.27	38.66	20.0	0.51 (0.38–0.67)	1.54 (35.7%)	4.27	31.6	43.33
ML LMR	0.12 (0.04–0.24)	1.38 (87.6%)	3.83	66.19	3.33	0.24 (0.13–0.4)	1.53 (74.9%)	4.23	52.63	6.67
ML MHR	0.44 (0.31–0.6)	1.35 (42%)	3.75	37.67	20.0	0.53 (0.39–0.68)	1.15 (33.5%)	3.19	30.48	50

The SEM% and CV values reflect similar results to the lowest values for the path length. CVCR negatively correlates with the CV, but in some cases (e.g., the path length) shows that while the mean CV is way below the arbitrary cutoff value of 0.3, still not every subject could meet this criterion of CVCR < 100% (Table 2).

## Discussion

The reliability of eleven CoP parameters was studied on healthy young individuals. The novelty of the study is that not only bipedal stances with eyes open or closed conditions were tested in 30- and 60-second trials but also a single leg stance on the dominant leg was tested as well. A new reliability parameter, CVCR, was additionally used to assess the reliability. The results show that the path length yielded consistently the highest reliability, while the LDD and CE area yielded consistently fair-to-good reliability. A difference could be observed in the LA parameters, where the 60-second sampling interval provided fair-to-good reliability in every stance type, while poor reliability was achieved in 30-seconds measurements. A comparison between our results and other studies that addressed the reliability of common CoP parameters is presented in Table 5, which indicates similar results between studies.

**Table 5:** Comparison of reliability (ICC) of common CoP parameters with the literature.

	Population (sample size)	Sampling	EO				EC			SL		
			CE area	Path length or mean velocity	Maximum velocity	MPF (AP/ML)	CE area	Path length or mean velocity	MPF (AP/ML)	CE area	Path length or mean velocity	MPF (AP/ML)
[17]	Healthy young adults (10)	30 s	0.61 (0.08–0.89)	0.82 (0.57–0.92)	0.79 (0.45–0.94)							
[16]	Healthy young adults (12)	60 s	0.4	0.53		0.46 / 0.53	0.43	0.44	0.46 / 0.43			
[4]	Healthy adults (12)	20 s		0.78				0.78				
[30]	Young adults (30)	3x30 s averaged								0.85	0.72 / 0.75 AP /ML	0.68 / 0.7 AP/ML
[18]	Healthy young adults (16)	60 s						0.86 (0.72-) / 0.91 (0.81-) (AP/ML)				
[43]	Healthy young adults (44)	51.2 s		0.768 (0.647-)				0.773 (0.653-)				
[19]	Healthy adults (21)	60 s	0.859 (0.749–0.934)	0.710 (0.528–0.855)			0.960 (0.923–0.982)	0.879 (0.781–0.944)				
[11]	Hip osteoarthritis patients (38)	54 s	0.52 (0.08–0.8)	0.85 (0.64–0.95)	0.76 (0.44–0.91)	0.44 (-0.04–0.76) / -0.4 (-0.8–0.15)						
[10]	Spinal cord injury patients (23)	51.2 s	0.64	0.89			0.94	0.92				
[12]	Knee osteoarthritis patients (25)	3x10 s								0.54 (0.13–0.79)	0.87 (0.70–0.95)	
[13]	Healthy elderly people (7)	30 s, 60 s, 120 s	0.22, 0.47, 0.41	0.73, 0.77, 0.83		0.34, 0.09, 0.44						
[14]	People with musculoskeletal disorder (33)	30 s	0.33 (0.00–0.60)	0.84 (0.70–0.92)			0.64 (0.38–0.81)	0.91 (0.82–0.95)				
[15]	Healthy (22) / Post-stroke (20)	10 s	0.63 (0.29–0.83), 0.35 (-0.09–0.67) / 0.52 (0.13–0.77), 0.98 (0.95–0.99)	0.91 (0.8–0.96), 0.94 (0.98) / 0.82 (0.61–0.93), 0.82 (0.6–0.93)								
Current study	Healthy young adults (30)	30 s, 60 s	0.39 (0.26–0.56), 0.43 (0.3–0.6)	0.67 (0.54–0.79), 0.75 (0.64–0.85)	0.43 (0.3–0.6), 0.13 (0.05–0.26)	0.27 (0.16–0.44), 0.33 (0.21–0.5) / 0.22 (0.12–0.37), 0.3 (0.18–0.47)	0.42 (0.29–0.59), 0.39 (0.26–0.56)	0.71 (0.6–0.82), 0.74 (0.63–0.84)	0.42 (0.29–0.58), 0.43 (0.3–0.6) / 0.43 (0.3–0.6), 0.4 (0.27–0.57)	0.58 (0.45–0.72), 0.5 (0.36–0.66)	0.73 (0.62–0.83), 0.82 (0.73–0.89)	0.24 (0.13–0.39), 0.44 (0.3–0.6) / 0.46 (0.33–0.62), 0.55 (0.41–0.7)

CE: 95% Confidence ellipse, EO: eyes open bipedal stance, EC: eyes closed bipedal stance, SL, eyes open single leg stance, MPF: mean power frequency, AP: anteroposterior direction, ML: mediolateral direction. EC and SL maximum velocity is not compared because it is not presented in any other studies. There were no studies to compare to for the EC and SL maximum velocity.

Few researchers studied the reliability improvement of CoP parameters by averaging several measurements [12,13]. Although higher ICC values could be achieved by averaging, if the original standard deviation for the single measures is too large, the responsiveness for balancing alterations of the averaged parameter could degrade. Furthermore, the performance of repeated measurements is not always an option since time constraints might not allow it in clinical conditions, or it could be too exhausting for elderly people and people with impaired balance. Therefore, the averaging of the CoP parameters was not an option considered in this study.

The SEM and MDC values were consistent with the ICC values (Tables 2–4). The main focus was on the relative SEM% values for relative evaluation and inter-parameter comparison since the parameters often have different dimensions. The smallest relative standard measurement errors were found for the path length in each stance type in conjunction with the ICC. The acceptance limit for SEM% (<15%) defined by Laroche et al [11] were not achieved for most of the parameters that were achieved in their study. This difference in the number of accepted parameters might be due to the deviations in the measurement procedure, because they downsampled the CoP data to 40 Hz before processing, while in the current study, 100 Hz was used. This difference could significantly influence the CoP parameters, mostly the frequency type parameters. Only the SL path length parameter achieved an SEM% that was lower than the limit defined in [11]. The explanation for this finding is that in the SL stance, the path length is significantly longer compared to the bipedal stances due to the increased postural sway. Because SEM% is the ratio of SEM and the mean, the otherwise similar standard deviations are relatively smaller compared to the increased mean, which results in smaller SEM% values.

The SL stance is an important test method in the analysis of the effect of the foot structure on the postural stability [27–29]. The SL stance could be truly useful in detecting better-than-average postural control, e.g., in research studies on sportsmen. On the other hand, the SL stance is often non-feasible for elderly and disabled people in clinical diagnostics.

Usually, the frequency type parameters yielded poor reliability. As described by [26], the frequency of the largest amplitudes in the CoP motions is inconsistent. However, these large motions will result in the highest power frequency bins in the spectrum. If the frequency of these high-power frequency bins is inconsistent, then the resulting frequency-based CoP parameters yield large scattering in the values.

Most types of time-distance CoP measures can be evaluated in a simple manner: the larger the value is, the worst the balancing capability (CE area, path length, maximum velocity, anterior and posterior maximum deviation, LA). Although in some situations more stable persons might be able to afford more movement, instable persons control their movements more tightly. Ratio-type parameters are interpreted on a different scale and describe the orientation of the sway. These could be useful in clinical measurements when the monitored therapy affects the sway orientation or laterality. Otherwise, these are not recommended in cases where only simple and highly reliable assessments of the balancing quality are required. Anterior and posterior maximum deviations and AP and ML LA are extreme value parameters, just as the AP-ML range ratio is the ratio of the CoP path extremes. As such, they usually show poor reliability, but they are important to study because they can reflect the possibility of falling, which is an important field of study–especially in the elderly and in osteoarthritis patients.

Although LDD is not a CoP-based parameter, it is important, and it consistently showed fair-to-good reliability. Its usage can highlight the differences in the conditions of diminished limb health, especially in cases that involve the monitoring of unilaterally involved problems [8,9].

The present study might play a role in planning future stabilometry studies, because the reliability of several time-distance and frequency-based CoP parameters were studied on young, healthy individuals not only in bipedal stances but also in a single leg stance for 30- and 60-second measurement intervals. The limitations of the study are the lack of external validity to aged or diseased populations and the lack of analysis of factors such as sampling frequency or filter design.

## Conclusions

The present study estimated the reliability of a large set of time-domain and frequency domain CoP parameters that were calculated for both bipedal and single leg stances in 30- and 60-second measurement intervals. The repeatability of the parameters was calculated from ten trials in each stance type. The results show that the path length is the most reliable parameter, while the LDD, CE area and LA parameters are moderately reliable. Further stabilometry studies could consider these findings in the design of the study to properly characterize the postural stability. It is suggested that only the simplest and most reliable parameters should be used for clinical diagnostic and rehabilitation monitoring; however, maximum deviations and largest amplitude parameters are also good indicators of the risks of falling.

## Supporting information

S1 File Individual measurement data. (XLSX)
